# Impact of trehalose and hydroxychloroquine on the function of BRAF (V600E)-siRNA in the A375 melanoma cells

**DOI:** 10.22038/ijbms.2025.85017.18394

**Published:** 2025

**Authors:** Elmira Toobchi, Rana Moradian Tehrani, Mohammadreza Sharifi, Fatemeh Tabandeh, Seyedeh Negin Hadisadegh

**Affiliations:** 1 Department of Genetics and Molecular Biology, Faculty of Medicine, Isfahan University of Medical Sciences, Isfahan, Iran; 2 Department of Biophysics, Faculty of Biological Sciences, Tarbiat Modares University, Tehran, Iran

**Keywords:** BRAF (V600E), Hydroxychloroquine, Melanoma, siRNA, Trehalose

## Abstract

**Objective(s):**

Melanoma, a lethal form of skin cancer, is closely linked to mutations in melanocytes. Due to increased resistance to chemotherapy, innovative strategies, including gene and combination therapies, are being explored. This study evaluates the effects of trehalose (TRE) and hydroxychloroquine (HCQ) in enhancing the efficacy of BRAF (V600E)-siRNA in A375 cells.

**Materials and Methods:**

The A375 cells were treated, and changes in cell viability were assessed using MTT assays. Apoptosis was evaluated using flow cytometry. Additionally, gene expression analysis of B-Raf proto-oncogene, serine/threonine kinase (BRAF), Caspase 3 (CASP3), and Phosphoinositide-3-kinase regulatory subunit 3 (PIK3R3) was performed using quantitative RT-PCR.

**Results:**

HCQ treatment reduced cell viability and increased apoptosis compared to both control cells and cells treated with TRE. Gene expression analysis showed a significant down-regulation of BRAF expression in cells treated with HCQ and BRAF (V600E)-siRNA compared to siRNA-only treated cells. CASP3 expression was significantly up-regulated in cells treated with combined HCQ and siRNA, indicating a stronger apoptotic response. PIK3R3 expression showed no significant change in the transfected groups.

**Conclusion:**

TRE, either alone or combined with siRNA, showed limited efficacy and may counteract the apoptotic benefits of HCQ. Conversely, HCQ, whether used alone or in combination with siRNA, enhanced apoptosis, suggesting promise as a potential treatment for melanoma.

## Introduction

Melanoma is the most severe type of skin cancer, arising from the malignant transformation of melanocytes, which originate from neural crest cells responsible for melanin synthesis (1). Although melanoma accounts for only 5% of all skin cancers, its incidence is steadily increasing (2). The standard therapeutic approach to melanoma primarily relies on single-agent chemotherapeutic treatments, such as 5-fluorouracil or cisplatin (3, 4). However, these chemotherapy regimens often yield suboptimal therapeutic responses (5, 6). A significant obstacle to the success of chemotherapy is its dose-dependent toxicity, which significantly limits its systemic application (7, 8). Advanced technologies, such as nanoliposomes and the delivery of siRNA via nanoparticles, have been developed to enhance the targeted delivery of chemotherapeutic agents to malignant cells. These approaches minimize the impact on healthy tissues while reducing the required dose and associated toxicity of chemotherapy (9).

Melanoma is associated with various genetic factors that contribute to its development. It can be classified into four subtypes based on tumor mutations: BRAF, Neuroblastoma RAS Viral Oncogene Homolog (NRAS), Neurofibromatosis Type 1 (NF1), and triple wild type (1). These genetic mutations serve as significant prognostic factors for the disease and its treatment (6, 10). Understanding the genetic factors that influence the development and progression of melanoma is essential for designing effective treatment strategies. Mutations in NRAS and BRAF are present in approximately 80% of the most common melanomas (5, 11). BRAF is a cytoplasmic serine-threonine kinase that plays a pivotal role in cellular processes such as proliferation and differentiation. Mutations in the BRAF gene are a common driver of various malignancies. Elucidating the molecular mechanisms underlying these mutations is crucial for developing targeted cancer therapies (8, 12). The most prevalent genetic alteration, observed in approximately 90% of cases, involves a T-to-A transversion at nucleotide 1799 (T1799A), which results in the substitution of glutamic acid (E) with valine (V) at position 600 (10). Numerous studies have demonstrated that siRNA targeting the BRAF gene can selectively silence melanoma cell lines expressing this mutation, both *in vitro* and *in vivo* (9, 12). This inhibition reduces the levels of mutant BRAF protein and leads to increased phosphorylation of Extracellular Signal-Regulated Kinase (ERK). These findings suggest that gene therapy using BRAF siRNA molecules may represent a promising avenue for future clinical trials (13).

Autophagy, a process involving the degradation and recycling of intracellular components, supplies materials for metabolic pathways and maintains cell survival during periods of starvation (14). The outcome of autophagy is stimulus-dependent and can lead to varying results, ranging from cell survival to cell death (15, 16). Additionally, a complex relationship exists between apoptosis and autophagy, with cancer cells exhibiting opposing responses when treated with autophagy modulators in conjunction with apoptosis inducers (17, 18). These effects can either sensitize cancer cells to treatment or provide protective effects. Currently, pharmacological manipulation of the autophagic process has been proposed as a strategy to alter the distribution, activity, and functionality of oligonucleotides (19). Among these approaches, 4-aminoquinoline compounds, such as chloroquine (CQ) and its derivative hydroxychloroquine (HCQ), originally used to treat malaria, have shown promise as repurposed anticancer agents. Building on this therapeutic potential, chloroquine interferes with lysosomal function and inhibits autophagy, thereby promoting tumor cell death. These mechanisms have been demonstrated in melanoma models and further supported by studies investigating novel chloroquine derivatives (20, 21).

Another autophagy modulator is the naturally occurring glucose disaccharide trehalose (TRE). Studies have demonstrated the effectiveness of TRE in suppressing the growth of A375 melanoma cells (22). Additionally, TRE has been shown to induce robust autophagy or senescence in these cells (23).

The objective of this study was to evaluate the impact of two autophagy modulators, TRE and hydroxychloroquine (HCQ), on the efficacy of BRAF(V600E)-siRNA in A375 melanoma cells containing the BRAF(V600E) mutation. To this end, BRAF(V600E)-siRNA was transfected into A375 cells, then administered either TRE, HCQ, or their combination. The effects on cell viability, apoptosis, and the relative expression levels of the BRAF, Caspase3, and PIK3R3 genes were then assessed.

## Materials and Methods

### Cell cultures

The human melanoma cell line A375 (obtained from the Pasteur Institute of Iran) was cultured in Roswell Park Memorial Institute (RPMI)-1640 medium (BIO-IDEA) supplemented with 10% heat-inactivated fetal bovine serum (FBS; BIO-IDEA) and 1% penicillin-streptomycin solution (PEN-STREP; BIO-IDEA). The cells were maintained at 37 °C in a humidified atmosphere containing 5% CO_2_ and were passaged 3 to 4 times before transfection.

### Cell transfection

*Preparation of BRAF (V600E)-siRNA and scramble*

For the silencing of BRAF (V600E), the siRNA designed in the study (12) was utilized. The sequences of the siRNA (siBraf-mu; targeting BRAFV600E) and the scramble control (non-targeting) were as follows:

• **siRNA**: antisense, 5′-AUC GAG AUU UCU CUG UAG Cdtdt-3′; sense, 5′-GCU ACA GAG AAA UCU CGA Udtdt-3′

• **Scramble**: antisense, 5′-UCU CUU GGC GAG ACU AUA Udtdt-3′; sense, 5′-AUA UAG UCU CGC CAA GAG Adtdt-3′ (Genecust).

According to the manufacturer’s instructions, 500 µl of Diethyl Pyrocarbonate (DEPC)-treated water was used to prepare a 20 µM stock solution of both BRAF(V600E)-siRNA and the scramble control. These stock solutions were stored at −20 °C.

*Confirmation of cell transfection*

A375 cells were cultured in two experimental groups, scramble and control, in a 96-well plate and incubated at 37 °C in a humidified atmosphere containing 5% CO_2_. After 24 hr, transfection was performed using a scrambled oligonucleotide labeled with Cy3 fluorescent dye at the 5′ end, following the instructions from the transfection kit. Transfection was performed using METAFECTENE® PRO (Biontex) transfection reagent. Each well was examined under a fluorescence microscope at 24, 48, and 72 hr post-transfection. Based on the microscopic analysis of fluorescent images and the duration of cell treatments with HCQ and TRE, the optimal time interval for transfection was determined and implemented.

*Transfection of cells by BRAF (V600E)-siRNA*

Cells were seeded in an appropriate plate based on the experimental requirements and incubated at 37 °C to allow for adhesion. After 24 hr, the medium was replaced with pure RPMI. Subsequently, a mixture of 1× SI+ buffer, METAFECTENE® PRO, and siRNA was added to the medium. Following four hours of incubation at 37 °C, antibiotics and fetal bovine serum (FBS) were introduced into the medium.

### MTT assay

To assess cell viability, 7 × 10³ A375 cells were seeded into a 96-well plate and incubated at 37 °C to allow adhesion. After 24 hr, the test groups were transfected and incubated under the same conditions. Subsequently, the medium in all wells was replaced, and all groups (except the control group) were treated with TRE (60 mM), HCQ (25 M), or a combination of both. The cells were then re-incubated for 72 hr.

Following incubation, the supernatant medium was removed from all wells, and 50 µl of RPMI medium was added to each well. Next, 50 µl of MTT solution was introduced into each well, followed by incubation for 3.5 hr. Afterward, the supernatant was discarded, and 150 µl of Dimethyl Sulfoxide (DMSO) was added to each well. Pipetting was performed thoroughly to ensure complete dissolution of formazan crystals in DMSO. The optical absorbance was measured at a wavelength of 570 nm using an ELISA reader.

### Assessment of apoptosis

A total of 2 × 10⁵ A375 cells were seeded into a 24-well plate and incubated overnight at 37 °C in a humidified atmosphere with 5% CO_2_. After 24 hr, transfection was performed, and cells were treated with TRE (60 mM), HCQ (25 M), or a combination of both. The plate was then returned to the incubator.

Following 72 hr of incubation, the culture medium was removed, and each well was washed with 600 µl of phosphate-buffered saline (PBS) (BIO-IDEA). Subsequently, 200 µl of trypsin (BIO-IDEA) was added to each well, and after three minutes of incubation at 37 °C, 1 ml of complete culture medium was added. The cells from each well were transferred to flow cytometry tubes.

Centrifugation was conducted at 1800 rpm for 10 min at 4 °C, and the supernatant was carefully discarded. The cell pellets were resuspended in 700 µl of PBS, and a second centrifugation was performed under the same conditions. After discarding the supernatant, 500 µl of 1× Binding Buffer was added to each tube, and the cells were resuspended to form a uniform suspension.

Next, 2 µl of Annexin V-FITC (fluorescein isothiocyanate) (Mahboub BioResearch Co.) was added to each tube, followed by 15 min of incubation at room temperature. Subsequently, 1 µl of propidium iodide (PI) (Mahboub BioResearch Co.) was added, and the tubes were incubated for an additional 1–5 min at room temperature. The prepared samples were then transferred to the flow cytometer for analysis.

### Relative quantification of BRAF, CASP3, and PIK3R3 gene expression

*RNA extraction*

To examine the relative gene expression levels using the real-time PCR method, RNA extraction was performed utilizing an extraction kit (Parstous). The RNA extraction procedure was conducted according to the manufacturer’s protocol. Table 1 includes information on gene names and their sequences from the NCBI database.

*cDNA synthesis*

cDNA synthesis was carried out using the Parstous kit, following the manufacturer’s instructions meticulously. PCR was performed using a Thermal Cycler (Bio-Rad) according to the specified temperature program. The reverse transcription conditions were as follows: 25 °C for 10 min, 47 °C for 60 min, 85 °C for 5 min, and 4 °C for storage. The resulting cDNA samples were stored at −20 °C.

### Gene expression evaluation by reverse transcriptionpolymerase chain reaction (RTPCR)

The primers used in the study were as follows:

• **GAPDH**: forward, 

5′-CTGACCCATACCCACCATCAC-3′; reverse, 

5′-ACAACCTTCTTGCAGCTCCTC-3′

• **BRAF**: forward, 5′-CAGGAAGAGGCGTCCTTAGC-3′; reverse, 5′-GAAGGAGACGGACTGGTGAG-3′

• **Caspase 3**: forward, 

5′-CATGGAAGCGAATCAATGGACT-3′; reverse, 

5′-CTGTACCAGACCGAGATGTCA-3′

• **PIK3R3**: forward, 

5′-GACTGGAGGGAGGTGATGATG-3′; reverse, 

5′-GAAGTCATTGGCTTAGGTGGC-3′

PCR was performed using the Mic Real-Time PCR System (Bio Molecular Systems) under the following conditions:

• **Initial denaturation**: 95 °C for 15 min (1 cycle)

• **Cycling**: 95 °C for 20 sec, 60 °C for 30 sec, and 72 °C for 30 sec (40 cycles per gene).

The reaction products were analyzed using 1.5% agarose gel electrophoresis, and GAPDH was used as the internal control.

### Statistical analysis

Data analysis was conducted using one-way ANOVA and *post hoc* analyses to determine statistically significant differences between groups. Tukey’s test was applied for pairwise comparisons of group means. All statistical analyses were performed using GraphPad Prism software (version 8; GraphPad Software, Inc., San Diego, CA, USA). A *P*-value of <0.05 was considered statistically significant. Unless otherwise specified, all assays were performed in triplicate.

## Results

### Cells were transfected with the Cy3-labeled scramble

**Figure 1:**
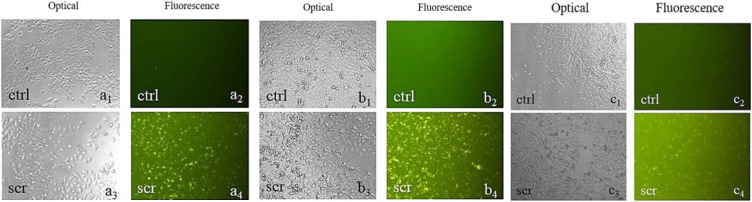
Optical and fluorescence microscopy images (×100) of A375 cells following Cy3-labeled scrambled siRNA transfection

Based on fluorescent microscope images, transfection was observed after 24 hr of incubation and reached its peak at 48 hr (Figure 1). Although the METAFECTENE® SI+ kit manual recommends 48 hr of incubation for optimal transfection, this study involved a 72-hr treatment of cells with TRE and HCQ. Consequently, a 24-hr incubation period was chosen for transfection instead.

### Combination of HCQ treatment and siRNA-mediated gene silencing reduced the viability of A375 cells

**Figure 2:**
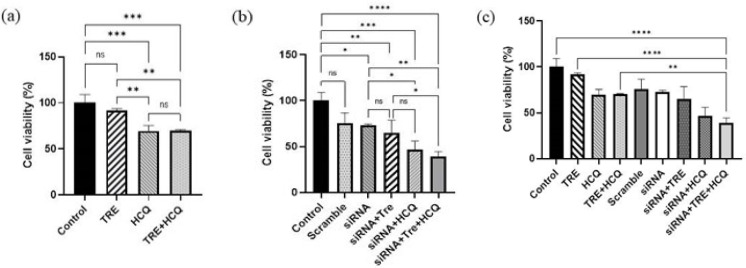
Evaluation of A375 melanoma cell viability following treatment with TRE (60 mM) and/or HCQ (25 µM), with or without BRAF (V600E)-siRNA transfection over a 72-hr period

To evaluate the effectiveness of various treatments on cell viability, an MTT assay and subsequent data analysis were performed. The results indicated no significant difference in cell viability between the group treated with TRE and the control group. However, treatment with HCQ resulted in a substantial reduction in cell viability compared to the control group (*P*<0.001). When the cells were treated with both TRE and HCQ, the percentage of cell viability decreased significantly compared to both the control group and the group treated with TRE alone (*P*<0.001). It is important to note that there was no significant difference in cell viability between the group treated with both compounds and the group treated only with HCQ (Figure 2a).

Analysis of the transfected groups revealed that the percentage of cell viability was significantly reduced in the siRNA-transfected group, as well as in the groups that underwent both transfection and simultaneous treatment, compared to the control group. Furthermore, the group treated with HCQ exhibited a significant decrease in the percentage of viable cells when compared to the transfected group without treatment. Notably, the group that was both transfected and treated with TRE and HCQ showed the lowest percentage of cell viability (*P*<0.0001). However, there was no significant difference in cell viability between this group and the transfected group treated solely with HCQ (Figure 2b).

Overall, the MTT assay results across all groups demonstrated that transfection significantly reduced the percentage of viable cells. Additionally, HCQ treatment, both in the presence and absence of transfection, caused a significant reduction in cell viability (Figure 2c).

### Combination of HCQ treatment and siRNA-mediated gene silencing increased apoptosis in A375 cells

To evaluate the effect of autophagy inhibition on apoptosis induced by siRNA-mediated gene silencing in the A375 melanoma cell line, cells were treated with HCQ alone, TRE alone, or a combination of both, with or without BRAF(V600E)-siRNA transfection. The analysis used an Annexin V-FITC/PI detection kit and flow cytometry. The percentages of apoptosis in the different groups were analyzed using FlowJo V10 software, and the results were graphed with GraphPad Prism 8 software.

**Figure 3:**
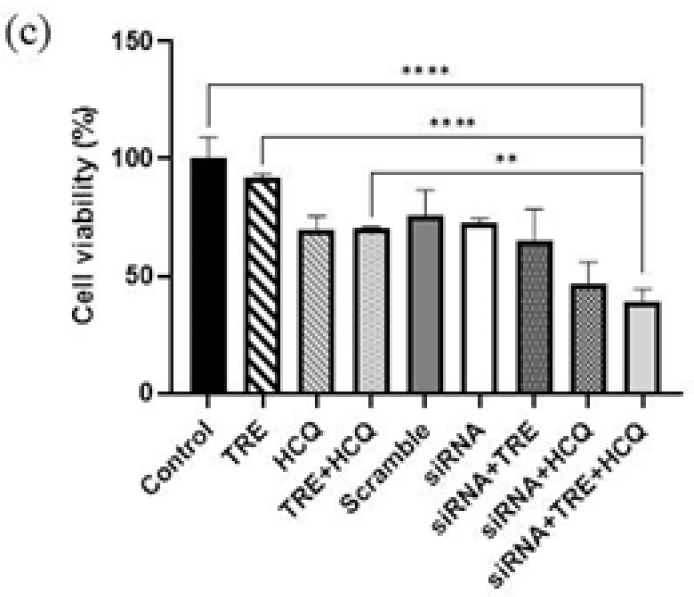
Effect of HCQ and TRE on apoptosis in A375 melanoma cells after 72 hr of treatment

The results showed that treatment with HCQ alone significantly increased the percentage of apoptosis compared to the control group. Conversely, treatment with TRE, either alone or in combination with HCQ, had no significant effect on the rate of apoptosis (Figure 3).

**Figure 4:**
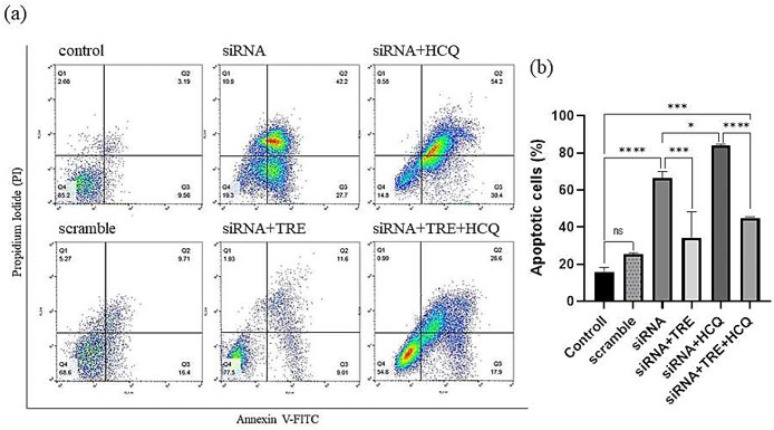
Effect of TRE and HCQ on apoptosis induced by BRAF (V600E)-siRNA transfection in A375 melanoma cells

In the siRNA-transfected groups, apoptosis increased compared to non-transfected controls. This increase was more pronounced in the group treated with HCQ alone after transfection. The group subjected to simultaneous transfection and treatment with both TRE and HCQ also exhibited increased apoptosis compared to the control group. However, the level of apoptosis in this group was lower than that observed in the group treated only with HCQ and the group that was solely transfected without additional treatment (Figure 4).

### Combination of HCQ treatment and siRNA-mediated gene silencing led to an increase in Caspase 3 gene expression in A375 cells

**Figure 5:**
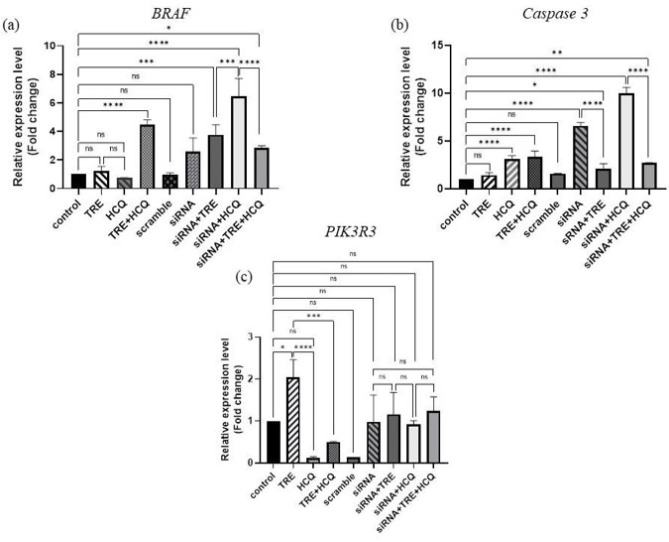
Impact of TRE and HCQ on siRNA-mediated gene silencing in A375 melanoma cells

To investigate the effects of combined treatment with TRE and HCQ, alongside BRAF(V600E)-siRNA transfection, on the A375 melanoma cell line, the expression levels of BRAF, CASP3, and PIK3R3 genes were analyzed using qRT-PCR (Figure 5).

The findings revealed that the combination of TRE and HCQ significantly increased BRAF gene expression (*P*<0.0001), whereas treatment with either compound alone did not affect its expression. Unexpectedly, BRAF gene expression in the siRNA-transfected group did not differ significantly from the control group. A significant increase in BRAF expression was observed in transfected groups treated with either TRE or HCQ, with the highest expression noted in cells treated with HCQ alone following transfection (*P*<0.0001). However, when comparing the group exposed to all three factors to the group that was only transfected, the combination of TRE and HCQ did not significantly enhance siRNA performance.

Regarding Caspase 3 (CASP3) gene expression, no significant changes were observed in cells treated with TRE. In contrast, treatment with HCQ markedly increased Caspase 3 expression (*P*<0.0001). This increase was also observed in cells treated with the combination of TRE and HCQ, although the combination did not result in any additional increase compared to HCQ treatment alone.

Transfection with siRNA significantly elevated Caspase 3 expression relative to the control group. Furthermore, HCQ treatment following siRNA transfection induced a significant increase in Caspase 3 expression (*P*<0.0001). Conversely, TRE treatment after transfection significantly reduced Caspase 3 expression compared to the group transfected with siRNA alone (*P*<0.0001). Nevertheless, TRE-treated transfected cells still showed a significant increase in Caspase 3 expression compared to the control group (*P*<0.05).

For PIK3R3 gene expression, TRE treatment led to a significant increase (*P*<0.05). However, HCQ treatment alone or combined with TRE did not significantly affect PIK3R3 expression. Comparing the siRNA-transfected groups among themselves and with the control group revealed no notable differences in PIK3R3 expression levels.

## Discussion

While invasive melanomas constitute only about 1% of cases, they account for over 75% of deaths related to skin cancer (24).  The limited efficacy of chemotherapeutic agents and the intrinsic resistance of melanoma cells are key factors contributing to the low response rates of current treatment modalities, which remain a significant challenge in melanoma therapy (25). In addition to traditional treatments like chemotherapy, newer options such as gene therapy are advised for melanoma patients. Advances such as RNA interference (RNAi) have enabled gene silencing through the use of siRNA (26). Half of cutaneous melanoma cases involve BRAF mutations, which drive cancer growth via mitogen-activated protein kinase (MAPK) pathway activation. Targeted siRNA therapy shows potential for silencing BRAF and advancing melanoma treatment (27, 28). 

Autophagy regulates cell survival or death depending on the stimulus intensity. It interacts with apoptosis, showing contradictory effects when cancer cells are exposed to modulators (29). Drugs modifying autophagy enhance oligonucleotide delivery and efficacy (30-32). CQ and HCQ, anti-malarial drugs, inhibit autophagy and show anticancer potential due to selective tumor targeting (33). TRE, an autophagy inducer, controls A375 melanoma cell growth and induces autophagy or senescence (22).

This study evaluated the effects of HCQ and TRE, two autophagy modulators, on siRNA-mediated gene silencing. A375 melanoma cells were transfected with BRAF(V600E)-siRNA and subsequently treated with TRE, HCQ, or their combination.

According to MTT assay results, treatment of A375 cells with TRE did not significantly affect their viability. This observation is consistent with the findings of Giulia Allavena and her team, who reported similar effects of TRE on cell viability in both A375 and SK-Mel-28 cell lines, as assessed through LDH secretion measurements. TRE influences cellular processes based on apoptotic susceptibility: it enhances autophagy in apoptosis-prone cells, thereby preventing cell death, and induces premature senescence in apoptosis-resistant cells, halting proliferation rather than causing cell death (22). Treatment with HCQ resulted in a significant reduction in cell viability, consistent with previous findings (30). Notably, the combination of TRE and HCQ resulted in a significant decrease in cell viability compared to the control group. However, when compared to cells treated exclusively with HCQ, no significant difference in survival rates was observed. 

According to a study conducted by He *et al*., the use of siRNA to inhibit the BRAF (V600E) gene in the A375 cell line led to a significant reduction in cell viability (12). Similarly, our study demonstrated comparable outcomes, revealing that treatment with HCQ further enhanced the reduction in cell viability in transfected cells. These findings suggest that inhibiting autophagy may amplify the effects of gene silencing in A375 melanoma cells. Inhibition of autophagy disrupts cellular protective mechanisms, sensitizing A375 melanoma cells to siRNA-mediated gene silencing. Studies have shown that autophagy inhibition, such as through chloroquine (HCQ), enhances apoptosis and reduces cell viability by impairing stress-response pathways that support cancer cell survival (34, 35).

Flow cytometry revealed that TRE had no significant effect on apoptosis in A375 cells, while HCQ markedly increased apoptotic activity. The combination of TRE and HCQ did not enhance apoptosis.

Research demonstrated that treating EAC mice with TRE induced apoptosis (36). Similarly, Kudo *et al*. reported that TRE treatment increased the apoptosis rate in the MEWO human melanoma cell line, as indicated by flow cytometry analysis (37). However, our findings did not align with these studies, as we observed inconsistencies in our results. In contrast, a study revealed that treating the ESCC cell line with CQ inhibited autophagy and subsequently increased apoptosis rates (38). Consistent with their observations, our study also reported an increase in apoptosis.

The siRNA-transfected group exhibited a substantial increase in apoptosis compared to the control group, confirming that transfection had a significant impact on cell mortality in the A375 cell line. Furthermore, treatment with HCQ significantly enhanced apoptosis in transfected cells. In contrast, treatment with TRE alone or in combination with HCQ resulted in a decrease in apoptosis within transfected cells. This suggests that the up-regulation of autophagy by TRE may have mitigated the pro-apoptotic effects of siRNA. Similarly, inhibiting TRE-induced autophagy produced a comparable outcome. Consequently, it cannot be concluded that TRE enhances the efficacy of siRNA through mechanisms independent of autophagy. However, combining HCQ with siRNA significantly increased apoptosis in A375 cells.

Treatment with TRE or HCQ did not alter the relative expression of the BRAF gene in A375 cells. However, simultaneous treatment with TRE and HCQ increased the relative expression of the BRAF gene when GAPDH was used as the reference gene.

The assessment of BRAF relative expression in siRNA-transfected groups yielded unexpected results. Despite transfection, BRAF expression levels remained unchanged and showed no significant difference compared to the control group. To explore potential reasons for these findings, a review of previous studies was conducted. For instance, Al Hashmi *et al*. performed trench sequencing and RNA-seq analysis on fifteen melanoma cell lines. These methods produced inconsistent results in three cell lines, which were subsequently identified through sequencing. It was discovered that these cell lines harbored the V600E mutation, which RNA-seq did not detect. To ensure reproducibility, similar experiments were conducted on three additional cell lines: A375, SKMEL28, and PIG1. Both A375 and SKMEL28 cell lines carried the V600E mutation, while the PIG1 cell line was derived from normal epidermal melanocytes and lacked the mutation. qPCR analysis revealed that only the wild allele was expressed in the PIG1 cell line, whereas both wild and mutant alleles were present in A375 and SKMEL28 cell lines, with the wild allele being more abundant at both DNA and RNA levels. Interestingly, BRAF expression was found to be higher in the PIG1 cell line compared to the mutant cell lines, suggesting a regulatory mechanism that may suppress BRAF expression in the presence of the V600E mutation (39).

Studies on BRAF gene inhibition have demonstrated that utilizing siRNAs to target both the wild-type and mutant forms of BRAF can yield more favorable outcomes. For instance, Kumar *et al*. reported that inhibiting the BRAF (V600E) gene with the relevant siRNA reduced the survival and proliferation of melanoma cells under hypoxic conditions. Notably, a greater reduction was observed when both the wild-type BRAF and the V600E mutant were simultaneously inhibited (40). Similarly, HE *et al*. showed that the simultaneous inhibition of wild-type and mutant BRAF by siRNA significantly reduced the viability of A375 cells (12). The observed lack of reduction in BRAF expression within the transfected group in our study may be attributed to the presence and expression of the wild-type BRAF allele in the cells. This raises the question of why apoptosis increased significantly in the transfected cell group, despite the siRNA not reducing BRAF expression. It is important to note that while siRNAs are primarily designed to silence specific targets, they can also potentially affect unintended genes (41). One such mechanism contributing to the off-target effects of siRNA is translational repression (42). Research indicates that siRNAs can retain their silencing capacity even when they exhibit multiple mismatches to the target mRNA (41). The siRNA employed in our study likely targeted various gene transcripts, in addition to BRAF. While these transcripts displayed a query cover percentage below 100%, their E-values matched that of BRAF, the primary target. This suggests that additional genes may have been affected by the siRNA. Given the complexity of gene functions in cellular processes such as metastasis, proliferation, and apoptosis, pinpointing the exact mechanisms underlying the siRNA’s actions remains challenging. Therefore, it is plausible that the siRNA used in our study induced apoptosis in A375 melanoma cells through mechanisms unrelated to BRAF inhibition.

The qPCR analysis of CASP3 gene expression revealed no significant difference in the TRE-treated group compared to the control group. However, a marked increase in CASP3 expression was observed in the groups treated with HCQ or the combination of TRE and HCQ. The rise in CASP3 expression observed in all groups, except the TRE-treated group, aligns with the apoptosis assay results, which indicated that TRE had no impact on apoptosis rate or CASP3 expression.

The TRE-treated group demonstrated a significant increase in the relative expression of the PIK3R3 gene compared to the control. PIK3R3 functions as an intracellular lipid substrate exchanger, playing a pivotal role in various signaling pathways and regulating key physiological processes, including cell proliferation, apoptosis, migration, and invasion. Studies have shown that dysregulation of PIK3R3 is strongly linked to tumor cell migration and invasion, with elevated levels of PIK3R3 enhancing melanoma cells’ migratory and invasive capabilities (43). In a previous study, a TRE-based compound, 6,6’-dideoxy-6,6’-bis(2-hydroxybenzamide)-α,α-D-trehalose, was shown to effectively suppress the migration and invasion of MDA-MB-231 cells (44). Treatment of A375 cells with TRE in our study resulted in an increased relative expression of the PIK3R3 gene, which is closely linked to cancer cell migration. These findings imply that TRE derivatives may be more effective than TRE alone in suppressing the metastasis of A375 cells. Furthermore, in siRNA-transfected groups, no significant changes were observed in the relative expression of PIK3R3.

TRE appeared to diminish the effects of HCQ and siRNA, reducing their overall efficacy. This could be attributed to trehalose’s autophagy-inducing properties, which may counteract the autophagy-inhibiting mechanism of HCQ, thereby decreasing its pro-apoptotic impact. Similarly, trehalose might interfere with siRNA’s ability to disrupt specific pathways by promoting cellular homeostasis, thus mitigating its apoptotic effects (45, 46).

This study highlights the impact of autophagy modulation on BRAF(V600E)-siRNA efficacy in A375 melanoma cells, emphasizing the role of HCQ in enhancing apoptosis, whereas TRE appeared to attenuate HCQ’s effect. The findings align with previous studies showing that autophagy inhibition through HCQ enhances apoptosis and decreases melanoma cell viability, reinforcing its potential as an adjuvant therapeutic strategy (12, 47). Furthermore, the regulatory interplay between the PI3K and MAPK pathways underscores the complexity of autophagy-mediated survival mechanisms in melanoma, consistent with findings in other studies (48, 49). While the use of a single cell line (A375) is a limitation, similar trends have been reported in WM115 and M14 melanoma models, supporting the generalizability of these observations (50).Additionally, recent reports, including data referenced in registered patents and conference abstracts, have identified resistance to BRAF/MEK inhibitors such as encorafenib/binimetinib as a persistent challenge in the treatment of BRAF-mutant melanoma (51, 52). Our findings propose that combining siRNA therapy with autophagy inhibition may offer a novel approach to overcoming resistance mechanisms, warranting further validation through *in vivo* studies and broader molecular analyses.

**Table 1:** List of genes (Homo sapiens) and their corresponding sequence links retrieved from the NCBI database

Gene Name	Organism	Sequence link	Database
**GAPDH**	Homo sapiens	GAPDH Sequence	NCBI
**BRAF**	Homo sapiens	BRAF Sequence	NCBI
**CASP3**	Homo sapiens	CASP3 Sequence	NCBI
**PIK3R3**	Homo sapiens	PIK3R3 Sequence	NCBI

GAPDH: Glyceraldehyde-3-phosphate dehydrogenase; BRAF: B-raf proto-oncogene serine/threonine kinas; CASP3: Caspase-3 apoptosis-related cysteine peptidase; PIK3R3: Phosphoinositide-3-kinase regulatory subunit 3; NCBI: National center for biotechnology information

The figure presents cellular morphology and Cy3 fluorescence signal in control and scrambled groups at three time points: 24, 48, and 72 hr post-transfection. Panels a1 to a2 show optical and fluorescence images of untransfected cells in the control group at 24 hours, with no Cy3 signal detected. Panels a3 to a4 correspond to transfected cells in the scrambled group at the same time point, showing initial uptake and weak Cy3 fluorescence. Panels b1 to b2 represent control cells at 48 hours, lacking fluorescence signal and confirming absence of transfection. In contrast, panels b3 to b4 show transfected scrambled cells at 48 hr, exhibiting stronger Cy3 signal intensity. Panels c1 to c2 again reflect control cells at 72 hr with no detectable signal, while panels c3 to c4 highlight sustained Cy3 fluorescence in transfected scrambled cells, suggesting prolonged intracellular retention of the labeled siRNA.

scr: Scramble; ctrl: Control

Cell viability was assessed using the MTT assay across multiple experimental conditions. Panel (a) shows the effects of TRE and/or HCQ treatment on non-transfected cells. Panel (b) illustrates the viability of cells transfected with BRAF (V600E)-siRNA prior to exposure to TRE and/or HCQ. Panel (c) provides a comparative summary of all groups, highlighting the differential impact of siRNA transfection combined with pharmacological treatment.

TRE: Trehalose; HCQ: Hydroxychloroquine; siRNA: Small interfering RNA; ns, non-significant; *P< 0.1; **P< 0.01; ***P< 0.001; ****P< 0.0001

Apoptosis was evaluated using flow cytometry following exposure to TRE (60 mM) and/or HCQ (25 µM) for 72 hours. The data demonstrate that HCQ significantly enhances apoptosis in A375 cells, whereas TRE alone has no notable effect. The figure includes panel (a), a representative flow cytometry dot plot of apoptotic cell distribution, and panel (b), a bar graph quantifying the percentage of apoptotic cells across all treatment groups.

TRE: Trehalose; HCQ: Hydroxychloroquine; FITC: Fluorescein isothiocyanate; ns: Non-significant; ****P< 0.0001.

To evaluate the effects of TRE and HCQ on apoptosis induced by siRNA-mediated gene silencing, A375 cells were transfected with BRAF (V600E)-siRNA and subsequently treated with TRE (60 mM) and/or HCQ (25 µM) for 72 hr. Flow cytometry analysis revealed that treatment with HCQ increased apoptosis induced by siRNA. In contrast, treatment with TRE reduced apoptosis levels caused by siRNA. Moreover, the combination of siRNA with both TRE and HCQ also decreased apoptosis induced by siRNA. Panel (a) shows a representative flow cytometry dot plot of apoptotic cell distribution, while panel (b) illustrates a bar graph quantifying apoptosis levels across the transfected experimental groups.

TRE: Trehalose; HCQ: Hydroxychloroquine; FITC: Fluorescein isothiocyanate; siRNA: Small interfering RNA; ns: Non-significant; *P<0.1; ***P<0.001; ****P<0.0001.

The relative expression levels of BRAF, CASP3 (Caspase 3), and PIK3R3 were assessed using qRT-PCR in A375 cells treated with TRE (60 mM) and/or HCQ (25 µM), with or without BRAF(V600E)-siRNA transfection. Panel (a) shows that BRAF expression remained unchanged following treatment with TRE, HCQ, or siRNA alone; however, combined treatment with TRE and HCQ or HCQ alone in transfected cells led to an increase in BRAF expression levels. Panel (b) illustrates that CASP3 expression was upregulated by siRNA transfection and further enhanced by HCQ treatment in transfected cells. Panel (c) demonstrates that TRE treatment significantly increased the relative expression of PIK3R3, regardless of transfection status.

TRE: Trehalose; HCQ: Hydroxychloroquine; siRNA: Small interfering RNA; ns: Non-significant; *P<0.1; **P<0.01; ***P<0.001; ****P<0.0001.

## Conclusion

The findings demonstrated that treatment with TRE alone had no beneficial impact on apoptosis or the expression of genes associated with melanoma progression and metastasis. Instead, TRE up-regulated the expression of the PIK3R3 gene, which is linked to metastatic activity. Moreover, the combination of TRE with siRNA did not notably enhance its efficacy. Conversely, HCQ treatment significantly increased CASP3 expression, thereby promoting apoptosis and improving siRNA performance. However, HCQ did not influence metastasis, as it did not affect the expression of PIK3R3.

The combination of all three factors led to increased apoptosis and up-regulated CASP3 expression compared to the control group. However, a comparative analysis revealed that the combination of siRNA with HCQ was more effective than combining these two factors with TRE. Based on these findings, TRE, whether administered alone or alongside siRNA targeting BRAF, cannot be considered a viable treatment strategy for melanoma. Conversely, HCQ, either as a standalone treatment or in combination with siRNA, demonstrates potential for future therapeutic approaches to combat this disease.

## Declaration

We acknowledge the use of Copilot in enhancing the language and readability of this paper.
